# Squamous cell carcinomas escape immune surveillance via inducing chronic activation and exhaustion of CD8^+^ T Cells co-expressing PD-1 and LAG-3 inhibitory receptors

**DOI:** 10.18632/oncotarget.13228

**Published:** 2016-11-09

**Authors:** Ameet K. Mishra, Tanya Kadoishi, Xiaoguang Wang, Emily Driver, Zhangguo Chen, Xiao-Jing Wang, Jing H. Wang

**Affiliations:** ^1^ Department of Immunology and Microbiology, University of Colorado, Anschutz Medical Campus, Aurora, CO 80045, USA; ^2^ Department of Biomedical Research, National Jewish Health, Denver, CO 80206, USA; ^3^ Department of Pathology, University of Colorado, Anschutz Medical Campus, Aurora, CO 80045, USA

**Keywords:** squamous cell carcinoma, immune evasion, PD-1, LAG-3, Smad4 loss

## Abstract

Squamous cell carcinoma (SCC) is the second commonest type of skin cancer. Moreover, about 90% of head and neck cancers are SCCs. SCCs develop at a significantly higher rate under chronic immunosuppressive conditions, implicating a role of immune surveillance in controlling SCCs. It remains largely unknown how SCCs evade immune recognition. Here, we established a mouse model by injecting tumor cells derived from primary SCCs harboring *Kras^G12D^* mutation and *Smad4* deletion into wild-type (wt) or CD8^−/−^ recipients. We found comparable tumor growth between wt and CD8^−/−^ recipients, indicating a complete escape of CD8^+^ T cell-mediated anti-tumor responses by these SCCs. Mechanistically, CD8^+^ T cells apparently were not defective in infiltrating tumors given their relatively increased percentage among tumor infiltrating lymphocytes (TILs). CD8^+^ TILs exhibited phenotypes of chronic activation and exhaustion, including overexpression of activation markers, co-expression of programmed cell death 1 (PD-1) and lymphocyte activation gene-3 (LAG-3), as well as TCRβ downregulation. Among CD4^+^ TILs, T regulatory cells (Tregs) were preferentially expanded. Contradictory to prior findings in melanoma, Treg expansion was independent of CD8^+^ T cells in our SCC model. Unexpectedly, CD8^+^ T cells were required for promoting NK cell infiltration within SCCs. Furthermore, we uncovered AKT-dependent lymphocyte-induced PD-L1 upregulation on SCCs, which was contributed greatly by combinatorial effects of CD8^+^ T and NK cells. Lastly, dual blockade of PD-1 and LAG-3 inhibited the tumor growth of SCCs. Thus, our findings identify novel immune evasion mechanisms of SCCs and suggest that immunosuppressive mechanisms operate in a cancer-type specific and context-dependent manner.

## INTRODUCTION

Squamous cell carcinomas (SCCs) are cancers that derive from stratified epithelia present in the skin and the lining of other organs such as aerodigestive tract. SCC is the second commonest type of skin cancer [1]; moreover, about 90% of head and neck cancers are SCCs (HNSCC). In addition, SCCs can occur in diverse tissues and present with vastly different symptoms. Risk factors for SCC of the skin include sunlight exposure and immunosuppression [2]. UV-induced skin cancers frequently harbor RAS mutations [3, 4]. HNSCC can be induced by carcinogen exposure such as tobacco or alcohol use or mediated by human papilloma virus infection [5]. Skin SCCs and tobacco-related HNSCCs often harbor heterozygous loss of *Smad4*, and *Smad4* downregulation is an early event in SCC development [6–8]. Consistently, mice with the deletion of *Smad4* in stratified epithelia develop spontaneous SCCs in the skin, oral cavity, and forestomach [6, 9, 10]. Recent studies showed that combining *Kras*^G12D^ mutation and *Smad4* loss in keratin 15-expressing (K15^+^) stem cells resulted in rapid development of aggressive SCCs that are highly metastatic [11]. It has been shown that patients who receive solid organ transplants develop SCCs at a significantly higher rate, probably due to their chronic immunosuppressive condition [12, 13], thereby suggesting a role of immune surveillance in controlling SCCs. However, it remains largely unknown how SCCs evade immune recognition.

Components of both innate and adaptive immune system participate in cancer immune surveillance [14], yet, its underlying mechanism in SCCs is less well understood. Prior studies support the notion that tumor cells themselves can orchestrate the local immune responses within tumor microenvironment [15], for instance, by producing pro-inflammatory and immunosuppressive cytokines or factors, recruiting immune suppressive cells into the tumor, modulating the expression of checkpoint pathway components that restrain T-cell responses, or creating a tumor microenvironment that may functionally reprogram T regulatory cells (Tregs) and render them more suppressive compared to their peripheral counterparts [16–19]. On the other hand, studies also suggest that the common inhibitory mechanisms including FoxP3^+^ Tregs, programmed cell death 1 (PD-1)/PD-ligand 1 (PD-L1) axis or indoleamine-2,3-dioxygenase expression might be a part of negative feedback that is intrinsically triggered by immune responses, instead of being orchestrated by tumors [20]. For example, it was shown that the recruitment of Tregs in melanomas was in fact dependent on CD8^+^ T cell, which occurred after the CD8^+^ T cell infiltration instead of preceding it [20]; furthermore, the upregulation of PD-L1 on tumor cells is induced by CD8^+^ T cells in an interferon (IFN)-γ-dependent manner [20, 21]. IFN-γ can be produced by NK cells, CD4^+^ or CD8^+^ T cells, and it is one of the major cytokines that have anti-tumor effects [22, 23]. The dysregulation of anti-tumor immunity has been suggested previously using carcinogen-induced SCC model [15, 24]. However, it remains unknown how the interplay between tumors and immune cells influence the immune evasion mechanisms of SCCs. It would be of great interest to investigate whether immune evasion mechanisms operate differentially in the context of different types of cancers.

Immune checkpoints are pivotal in mediating immune evasion of cancers, thus, immunotherapies have been developed to block immune checkpoints [25–28]. To date, the most extensively investigated immune checkpoints include cytotoxic T-lymphocyte protein 4 (CTLA4) and PD-1, nevertheless, many other immune checkpoints and immune-activating receptors exist such as lymphocyte activation gene-3 (LAG-3), TIM-3, OX40 and 4-1BB that deserve more intense investigation [27]. PD-1 was discovered more than two decades ago [29], and its main functions include inhibiting the activation of effector T cells, controlling self-reactive T cells and promoting the generation of Tregs [30]. LAG-3 has been shown to negatively regulate cellular proliferation, activation, and homeostasis of T cells, in a similar fashion to CTLA-4 and PD-1 [31, 32]. In particular, LAG-3 is important for the suppressive functions of CD4^+^ Tregs in autoimmune responses [33], and for maintaining tolerance to self and tumor antigens via dampening the activity of antigen-specific CD8^+^ T cells [34]. Currently, CTLA-4 and PD-1 inhibitors have been approved for cancer immunotherapy in clinics while TIM-3 and LAG-3 inhibitors are being tested in clinical trials [26, 35, 36]. It remains unknown which one of these inhibitors would specifically target SCCs harboring common genetic mutations found in patients, such as RAS activating mutations or *Smad4* deletion.

In the current study, we employed a transplanted mouse model to determine the signature of immune profiling (SIP) of tumor infiltrating lymphocytes (TILs) in the SCCs caused by *Kras*^G12D^ mutation and *Smad4* loss [11]. Our results showed that both CD8^+^ and CD4^+^ TILs co-expressed inhibitory receptors, PD-1 and LAG-3, and dual blockade of PD-1 and LAG-3 significantly suppressed the tumor growth of SCCs. Among CD4^+^ TILs, Tregs were preferentially expanded, which was independent of the presence of CD8^+^ T cells. Taken together, our data suggest that immune evasion mechanisms appear to operate in a cancer type-specific and context-dependent manner. Our studies may have important implications in designing targeted immunotherapy for SCCs and suggest that SIP evaluation of TILs might be critical for therapy selection.

## RESULTS

### SCCs escape CD8^+^ T cell-mediated immune surveillance

**Figure 1:**
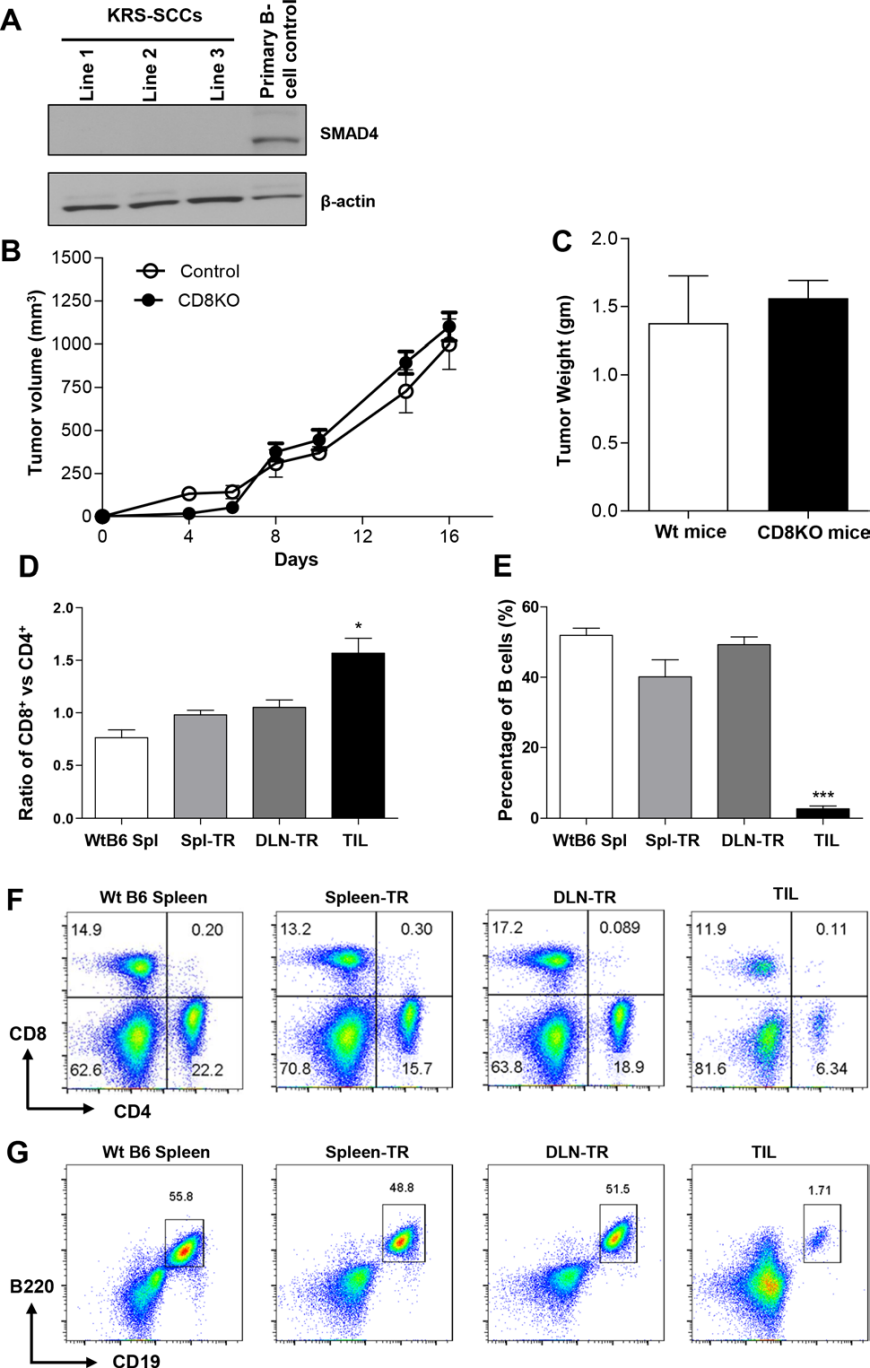
SCCs escape CD8+ T cell-mediated immune surveillance and immune profiling of TILs in KRS-SCCs (**A**) The absence of SMAD4 protein was confirmed in three KRS-SCC lines by western blot with primary B cells as positive control and β-actin as loading control. (**B**) KRS-SCC tumor cells were injected into wt or CD8^−/−^ recipients individually. Tumor volume was monitored in wt (Control, *n* = 12) and CD8^−/−^ recipients (CD8KO, *n* = 12). The tumor growth curve is not significantly different between two groups. (**C**) Tumor weight was measured at the end of experiments from = wt (wt mice, *n* = 12, white bar) or CD8^−/−^ recipients (CD8KO mice, *n* =12, black bar). Representative data are shown from four independent experiments. (**D**) Ratio of CD8^+^ vs CD4^+^ T cells in CD45^+^ population in different groups. WtB6-spl (*n* = 3), Spl-TR (*n* = 6), DLN-TR (*n* = 6) and TIL (*n* = 12). (**E**) Percentage of B cells in CD45^+^ population in different groups. WtB6-spl (*n* = 3), Spl-TR (*n* = 6), DLN-TR (*n* = 6) and TIL (*n* = 12). (**F** and **G**) Representative FACS plots are shown for CD8 vs CD4 (F) or B220 vs CD19 (G) staining in different groups. WtB6-spl: splenocytes isolated from wt naïve B6 mice; Spl-TR: splenocytes isolated from tumor recipients; DLN-TR: draining lymph node cells isolated from tumor recipients. TIL: tumor infiltrating lymphocytes. Data are presented as mean ± s.e.m. Data are representative results of 3 different cell lines in total 7 independent experiments for panel D-G (see details in Supplemental Table S1). Statistical significance was calculated with One-way ANOVA, Tukey's Multiple comparison test (Graphpad Version 5.01) among different groups, *P ≤ 0.05, ***P ≤ 0.001.

To elucidate the immune evasion mechanisms of SCCs, we established a transplanted tumor model using tumor cells derived from primary K15.Kras^G12D^.Smad4^−/−^ SCCs [11] (hereafter referred to as KRS-SCCs). Three tumor cell lines that have been passaged *in vivo* and *in vitro* were injected into wt C57BL/6 (B6) recipients (see details in Materials and Methods). The deletion of SMAD4 protein was confirmed in these cell lines via western blotting (Figure 1A). These KRS-SCCs evaded immune recognition and developed into secondary tumors in wt B6 immunocompetent recipients about two to four weeks after tumor inoculation. Given that CD8^+^ T cells play a dominant role in anti-tumor immunity, we next investigated whether the absence of CD8^+^ T cells affects the tumor growth by transplanting KRS-SCC tumor cells into CD8^−/−^ recipients. Surprisingly, we found that the tumor growth curve (Figure 1B) or the weight of tumors (Figure 1C) was not significantly different between wt and CD8^−/−^ recipients. These data suggest that CD8^+^ T cells in wt B6 recipients were grossly dysfunctional and did not play a significant role in the inhibition of tumor growth. We conclude that KRS-SCCs can evade CD8^+^ T cell-mediated immune surveillance and develop into secondary tumors.

### CD8^+^ T cells are capable of infiltrating KRS-SCCs with a preferential increase among TILs

To elucidate the mechanisms leading to the dysfunction of CD8^+^ T cells, we first examined whether CD8^+^ T cells were able to infiltrate tumors. We performed FACS analysis to characterize the signature of immune profiling (SIP) of TILs. Our results showed that both CD8^+^ and CD4^+^ T cells infiltrated the secondary KRS-SCCs while the percentage of CD8^+^ TILs was significantly higher than that of CD4^+^ TILs (Figure 1D, 1F). In contrast, the percentage of CD8^+^ T cells was relatively comparable (ratio~1.0) to that of CD4^+^ T cells in the spleen or draining lymph node (DLN) of wt B6 recipients inoculated with tumors (spleen-TR or DLN-TR), or even lower in the spleen of wt B6 mice without tumor inoculation (WtB6 Spl) (Figure 1D, 1F). We found that the percentage of tumor infiltrating B cells was much lower than that in other non-tumor site control groups, including WtB6 Spl, Spl-TR or DLN-TR (Figure 1E, 1G). Taken together, our data suggest that KRS-SCCs appear to be immunogenic evidenced with a preferential increase of CD8^+^ T cells at the tumor sites. Thus, we next focused on delineating how CD8^+^ TILs are dysregulated within the KRS-SCC tumor microenvironment.

### CD8^+^ TILs are overly activated and experience exhaustion manifested with co-expression of PD-1 and LAG-3

**Figure 2:**
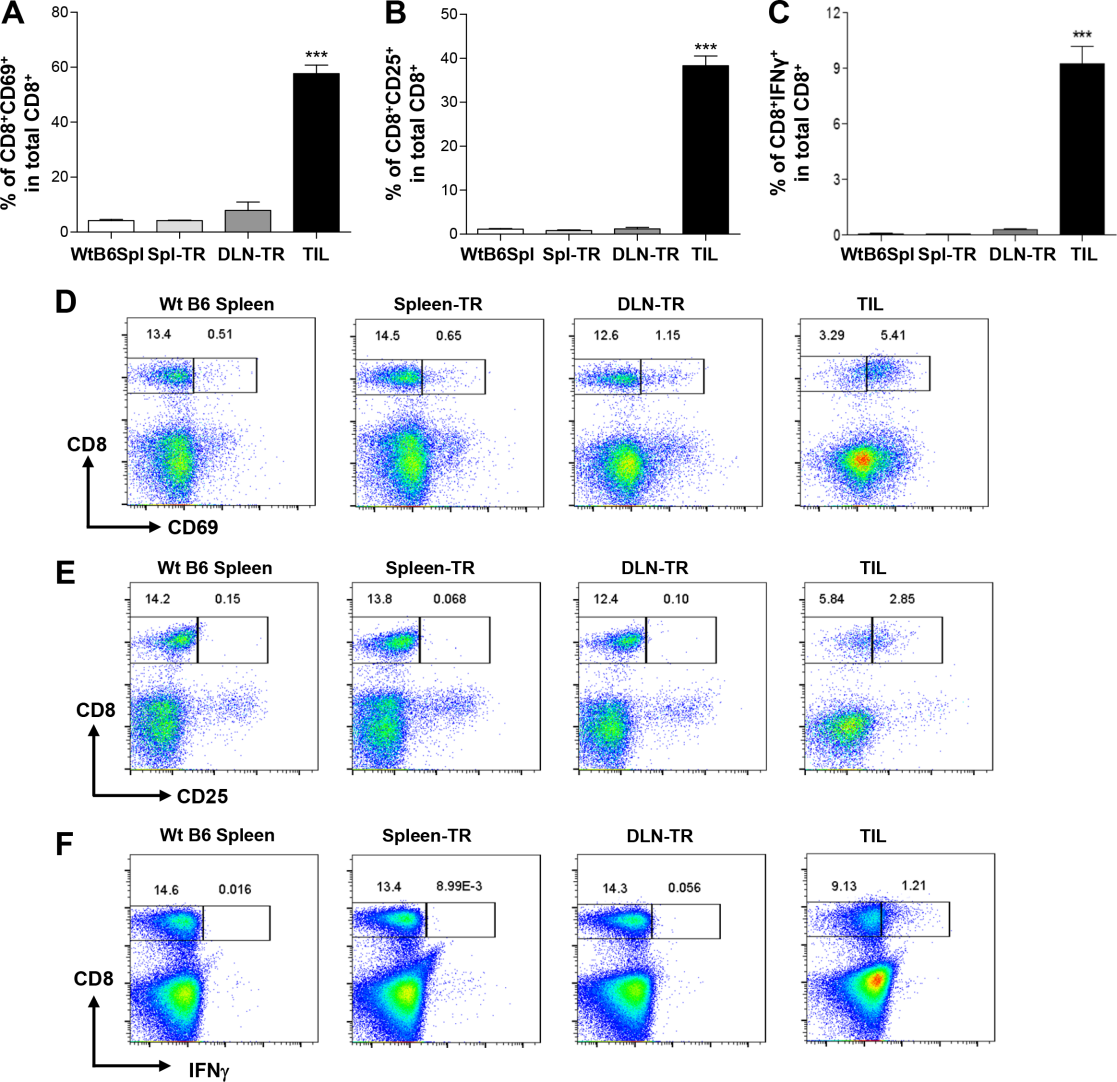
Upregulation of activation markers in CD8^+^ TILs (**A** and **B**) Percentage of CD8^+^CD69^+^ (A) or CD8^+^CD25^+^ (B) among total CD8^+^ T cells in different groups. WtB6-spl (*n* = 3), Spl-TR (*n* = 6), DLN-TR (*n* = 6) and TIL (*n* = 12). (**C)** Percentage of CD8^+^IFN-γ^+^ among total CD8^+^ T cells in different groups. WtB6-spl (*n* = 3), Spl-TR (*n* = 6), DLN-TR (*n* = 6) and TIL (*n* = 6). (**D**–**F**) Representative FACS plots are shown for CD8 vs CD69 (D), CD8 vs CD25 (E), or CD8 vs IFN-γ (F) staining in different groups. Data are presented as mean ± s.e.m. Data are representative results of five (A, B) or three independent experiments (C). Statistical significance was calculated with One-way ANOVA, Tukey's multiple comparison test, ***P ≤ 0.001.

To address why CD8^+^ TILs cannot prevent tumor growth, we characterized the phenotypes of CD8^+^ TILs. We found that CD8^+^ TILs significantly upregulated CD69 expression since the percentage of CD69^+^CD8^+^ T cells was remarkably higher in TILs than in other controls (Figure 2A, 2D). In line with these observations, we found that CD8^+^ TILs also significantly upregulated the expression of CD25, another activation marker for T cells (Figure 2B, 2E). Thus, our data suggest that CD8^+^ TILs exhibited the phenotypes of activated T cells. Next, we performed functional analysis of CD8^+^ TILs by examining their IFN-γ production with intracellular cytokine staining. Indeed, CD8^+^ TILs exhibited a modestly increased level of IFN-γ production compared to CD8^+^ T cells from other controls (Figure 2C and 2F). Thus, our data show that CD8^+^ TILs display the phenotypes and characteristics of activated T cells.

**Figure 3:**
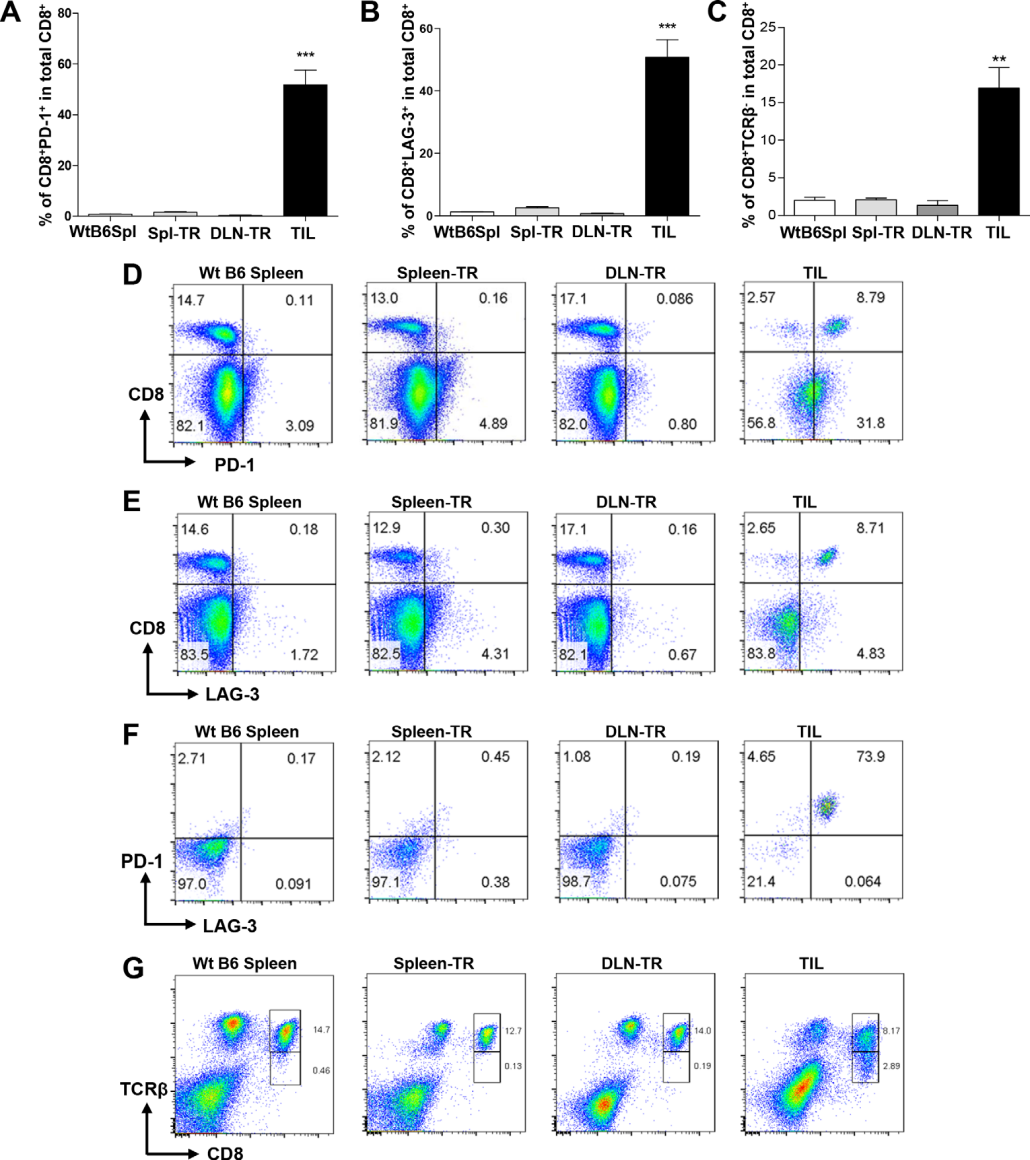
Co-expression of PD-1 and LAG-3 and downregulation of TCRβ in CD8^+^ TILs (**A** and **B**) Percentage of CD8^+^PD-1^+^ (A) or CD8^−^LAG-3^+^ (B) among total CD8^+^ T cells in different groups. WtB6-spl (*n* = 3), Spl-TR (*n* = 6), DLN-TR (*n* = 6) and TIL (*n* = 12). Data are presented as mean ± s.e.m. Representative data of 7 independent experiments are shown. (**C**) Percentage of CD8^+^TCRβ^−^ among total CD8^+^ T cells in different groups. WtB6-spl (*n* = 3), Spl-TR (*n* = 6), DLN-TR (*n* = 6) and TIL (*n* = 6). Data are presented as mean ± s.e.m. Representative data of 2 independent experiments are shown. (**D**–**G**) Representative FACS plots are shown for CD8 vs PD-1 (D), CD8 vs LAG-3 (E) PD-1 vs LAG-3 gated on CD8^+^ (F), CD8 vs TCRβ (G) staining in different groups. Statistical significance was calculated with One-way ANOVA, Tukey's multiple comparison test, ***P ≤ 0.001, **P ≤ 0.01.

Given that these apparently activated CD8^+^ TILs failed to reject tumors, we next tested whether these CD8^+^ TILs might also upregulate inhibitory co-receptors involved in suppressing T cell activation. We examined the expression of inhibitory co-receptors including CTLA-4, TIM-3, PD-1 and LAG-3 on CD8^+^ TILs. We did not detect any difference in the expression of CTLA-4 and TIM-3 on CD8^+^ TILs compared to other controls (data not shown). In contrast, we found that the percentage of PD-1^+^CD8^+^ T cells was strikingly increased in CD8^+^ TILs (Figure 3A and 3D). About 50–60% of CD8^+^ TILs expressed PD-1 on their surface whereas the percentage of PD-1^+^CD8^+^ T cells was rather minimal in other controls (Figure 3A and 3D). Apart from the upregulation of PD-1, we also detected a drastic increase of the percentage of LAG-3^+^CD8^+^ T cells in TILs as compared to other controls (Figure 3B and 3E). Notably, we found that a large fraction of CD8^+^ TILs co-expressed PD-1 and LAG-3, in contrast, such a population was completely absent in other controls (Figure 3F). Thus, our data showed that CD8^+^ TILs in KRS-SCCs specifically upregulated inhibitory co-receptors PD-1 and LAG-3. Consistent with the immune suppression phenotypes of CD8^+^ TILs, we found that the expression of T cell antigen receptor (TCR) β chain was downregulated in a larger percentage of CD8^+^ TILs compared to other controls (Figure 3C and 3G). Taken together, we conclude that CD8^+^ TILs have been overly activated and experienced exhaustion, which may contribute to the immune evasion of KRS-SCCs.

### CD4^+^ TILs showed a preferential expansion of Tregs

**Figure 4:**
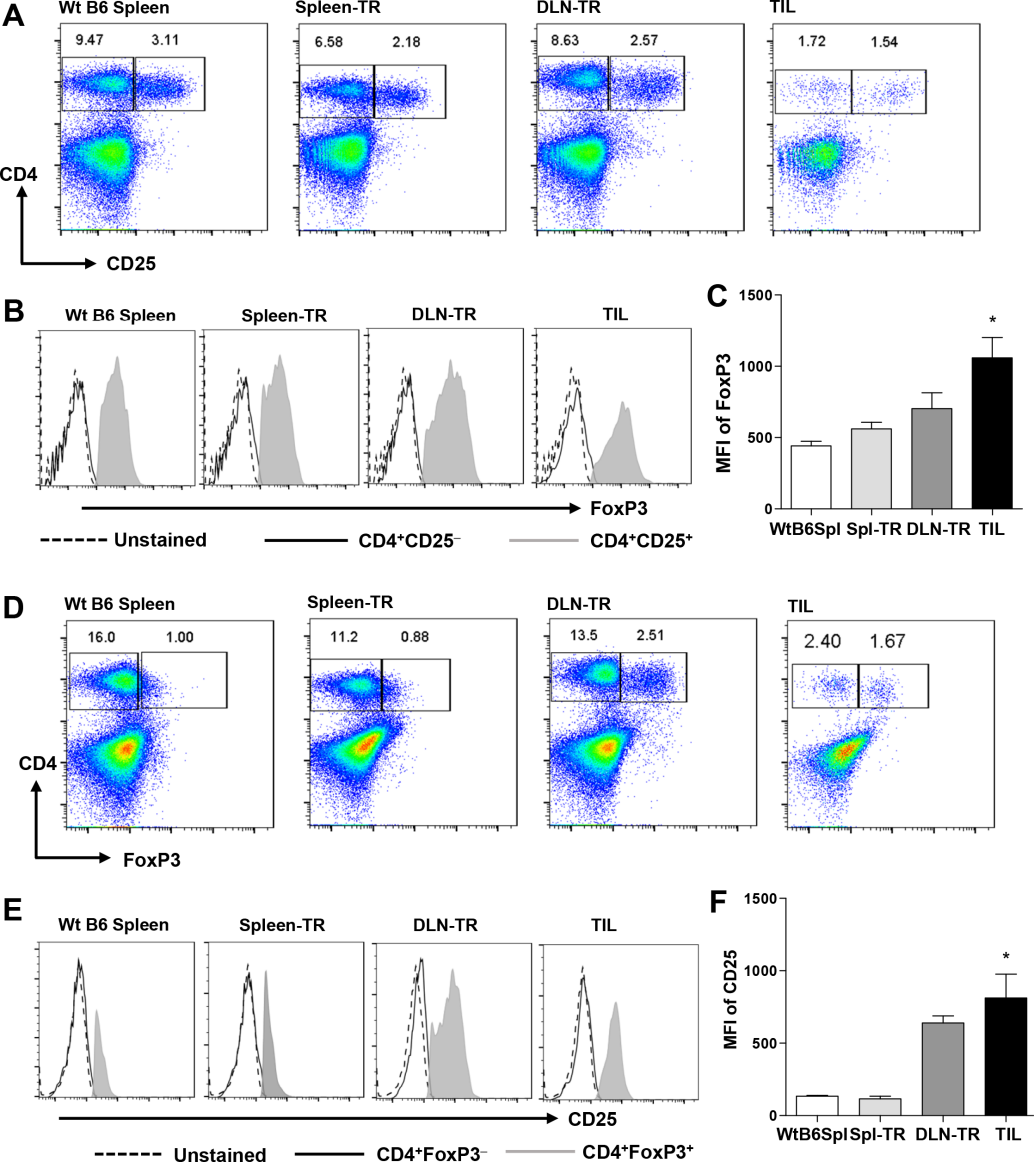
A preferential expansion of Tregs in CD4^+^ TILs Representative FACS plots are shown for CD4 vs CD25 (**A**) or CD4 vs FoxP3 (**D**) staining in different groups. Representative histogram plots are shown for FoxP3 (**B**) or CD25 (E) staining gated on different subsets of CD4^+^ T cells as indicated. (**C** and **F**) Mean Fluorescence Intensity (MFI) for FoxP3 staining in gated CD4^+^CD25^+^ cells (C), and for CD25 staining in gated CD4^+^FoxP3^+^ cells (F) in different groups. WtB6-spl (*n* = 3), Spl-TR (*n* = 6), DLN-TR (*n* = 6) and TIL (*n* = 12). Data are presented as mean ± s.e.m. Statistical significance was calculated with Student's *t-test* (Graphpad Version 5.01) between Spl-TR and TIL, *P ≤ 0.05. Data are representative results of 7 independent experiments.

We characterized CD4^+^ TILs and found that they did not significantly upregulate CD69 (data now shown). When we examined the expression of CD25 in CD4^+^ TILs, we identified two populations, CD25^−^ vs CD25^+^, and found that the CD25^+^CD4^+^ population was about 40% in CD4^+^ TILs, whereas this population was only about 15% in other controls (Figure 4A), suggesting an expansion of this population in CD4^+^ TILs. Next, we gated on the CD4^+^CD25^−^ or CD4^+^CD25^+^ population to determine the expression level of FoxP3 (Figure 4B), which is the lineage-specific transcription factor of Tregs. Our data showed that CD4^+^CD25^+^ TILs expressed a remarkably high level of FoxP3 while CD4^+^CD25^−^ TILs did not (Figure 4B). Notably, CD4^+^CD25^+^ TILs expressed a higher level of FoxP3 than other controls including WtB6 Spl and Spl-TR but not DLN-TR (Figure 4B and 4C). To further confirm these cells as Tregs, we also gated on the FoxP3^+^ vs FoxP3^−^ population in CD4^+^ TILs or other controls, and found that the percentage of CD4^+^FoxP3^+^ Tregs was much higher in TILs, which also appeared to be increased in DLN-TR group (Figure 4D). Consistently, CD4^+^FoxP3^+^ population expressed a much higher level of CD25 than CD4^+^FoxP3^−^ cells in all groups (Figure 4E). Notably, CD4^+^FoxP3^+^ TILs expressed a much higher level of CD25 than other controls including WtB6 Spl and Spl-TR but not DLN-TR (Figure 4E and 4F). Overall, our results showed a preferential expansion of Tregs in CD4^+^ TILs, which may serve as another mechanism to inhibit CD8^+^ TILs [37, 38].

### CD4^+^ TILs increased the expression of inhibitory co-receptors

**Figure 5:**
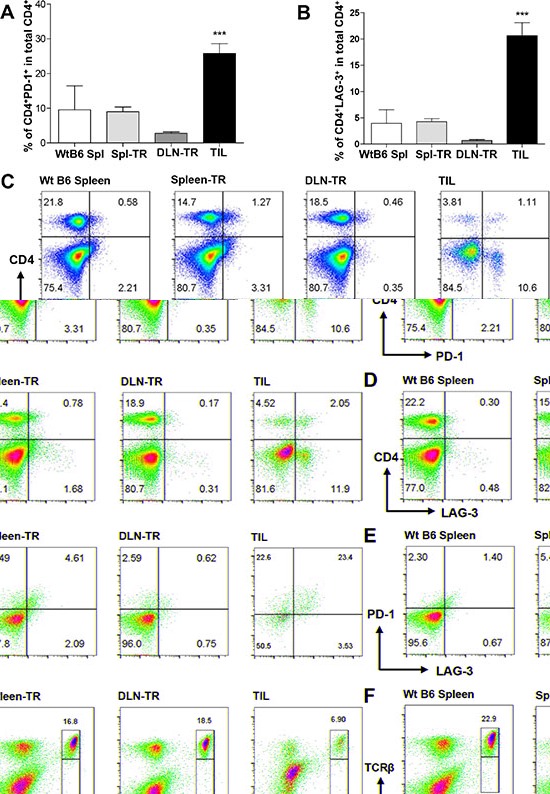
CD4^+^ TILs showed increased expression of PD-1 and LAG-3 but no change in TCRβ expression (**A** and **B**) Percentage of CD4^+^PD-1^+^ (A) or CD4+LAG-3+ (B) among total CD4^+^ T cells in different groups. WtB6-spl (*n* = 3), Spl-TR (*n* = 6), DLN-TR (*n* = 6) and TIL (*n* = 12). Data are presented as mean ± s.e.m. Representative data are shown from 7 independent experiments. (**C**–**E**) Representative FACS plots are shown for CD4 vs PD-1 (C), CD4 vs LAG-3 (D), or PD-1 vs LAG-3 (gated on CD4^+^ T cells) (E), CD4 vs TCRβ (**F**) staining in different groups. Statistical significance was calculated with One-way ANOVA, Tukey's multiple comparison test, ***P ≤ 0.001.

We examined whether CD4^+^ TILs also upregulated the inhibitory co-receptors as CD8^+^ TILs did. Our data showed that the percentage of CD4^+^PD-1^+^ appeared to be increased in TILs as compared with other controls (Figure 5A and 5C); though, such an increase was much less prominent than that of CD8^+^ TILs (Figure 3A and 3D). In addition, we detected a population of CD4^+^LAG-3^+^ in CD4^+^ TILs and the percentage of this population was much higher in TILs than in other controls (Figure 5B and 5D). Again, the increase of LAG-3^+^ population in CD4^+^ TILs was much less than that in CD8^+^ TILs (Figure 5B vs Figure 3B). Interestingly, while majority of CD8^+^ TILs co-expressed PD-1 and LAG-3, a relatively lower percentage of CD4^+^ TILs co-expressed PD-1 and LAG-3 (Figure 5E). Lastly, we examined whether CD4^+^ TILs also downregulated their TCRβ expression, in contrast to our findings of CD8^+^ TILs, CD4^+^ TILs did not alter their TCRβ expression compared to other controls (Figure 5F).

### The absence of CD8^+^ T cells affects other populations of TILs differentially

**Figure 6:**
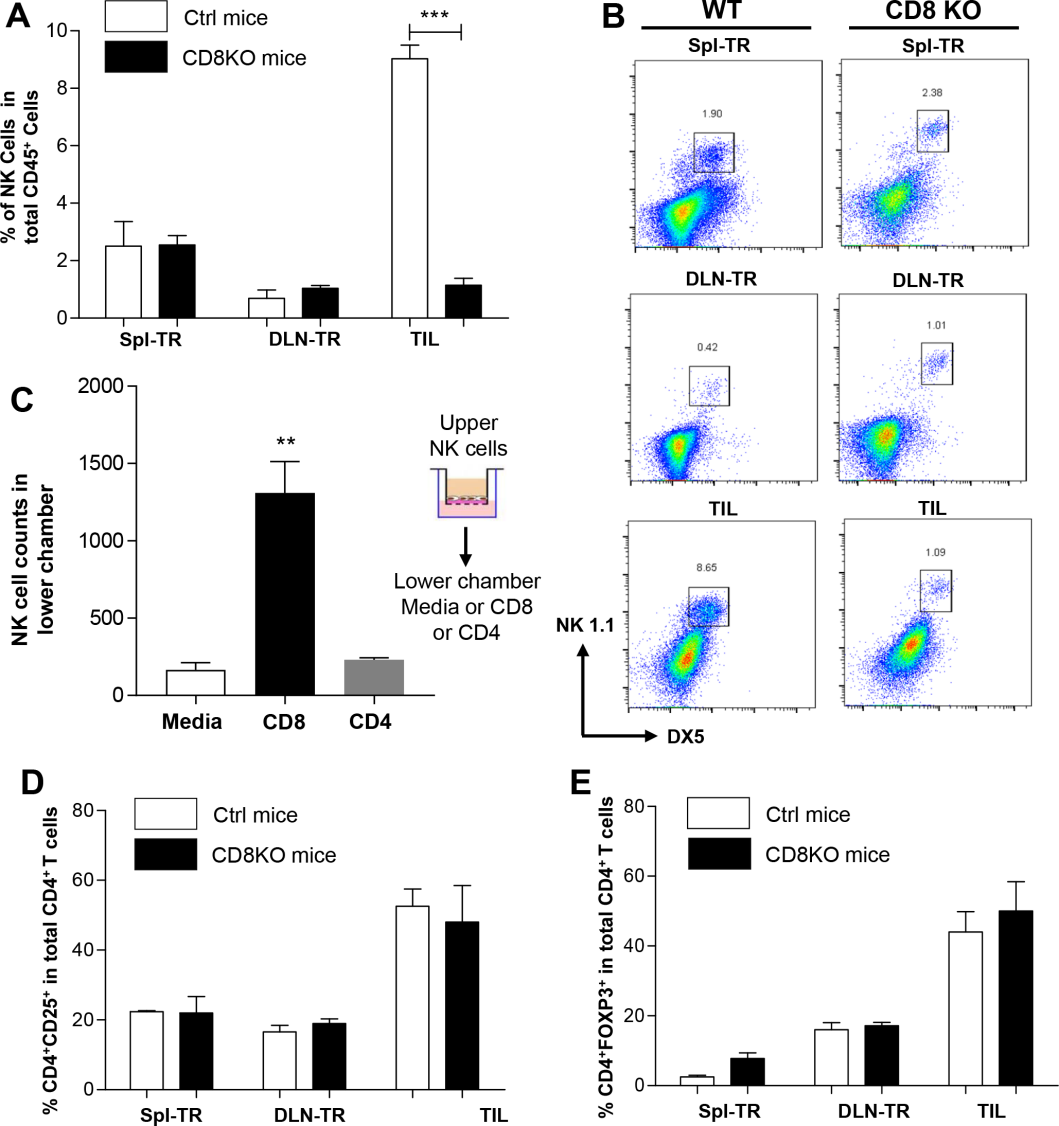
The impact of CD8^+^ T cells on other populations of TILs (**A**) Percentage of NK cells in CD45^+^ population in different groups of wt (white bar) or CD8^−/−^ (black bar) recipients. Spl-TR (*n* = 6), DLN-TR (*n* = 6) and TIL (*n* = 6). (**B**) Representative FACS plots are shown for NK1.1 vs DX5 staining in different groups of wt or CD8^−/−^ recipients. (**C**) Matrigel invasion assay. NK cells were seeded in upper chambers while lower chambers contained media only (*n* = 4), CD8^+^ T cells (*n* = 4) or CD4^+^ T cells (*n* = 4). The total number of NK cells invaded to lower chamber was calculated using flow cytometer. (**D** and **E**) Percentage of CD4^+^CD25^+^ (D) or CD4^+^FoxP3^+^ (E) among total CD4^+^ T cells in different groups of wt (white bar) or CD8^−/−^ (black bar) recipients. Spl-TR (*n* = 6), DLN-TR (*n* = 6) and TIL (*n* = 6). Representative data are shown from three independent experiments for panel A–E. Statistical significance was calculated with Student's *t-test* (Graphpad Version 5.01) between two groups as indicated, ***P ≤ 0.001, **P ≤ 0.01.

To investigate how the absence of CD8^+^ T cells affects the immune profiling of TILs, we transplanted KRS-SCCs into wt or CD8^−/−^ recipients. Compared to wt recipients, there was a remarkable reduction in the percentage of tumor infiltrating NK cells in CD8^−/−^recipients (Figure 6A and 6B). In contrast, the percentage of NK cells was comparable between wt and CD8^−/−^recipients in other control groups including Spl-TR and DLN-TR (Figure 6A and 6B), demonstrating a selective reduction of NK cells in TILs. Thus, we conclude that the increased percentage of tumor infiltrating NK cells is dependent on the presence of CD8^+^ T cells. To test whether CD8^+^ T cells affect NK cell migration directly, we employed an *in vitro* invasion assay. NK cells were plated in the upper chambers, while the lower chambers contained media or naïve CD8^+^ T cells or CD4^+^ T cells as control. Consistent with our *in vivo* data, we found that the number of NK cells migrating into the lower chamber was significantly increased when CD8^+^ T cells were present but not CD4^+^ T cells (Figure 6C).

Previous studies showed that the recruitment of Tregs in melanomas was dependent on CD8^+^ T cells, which occurred after the CD8^+^ T cell infiltration instead of preceding it [20]. Thus, we examined the effects of CD8^+^ T cells on Treg population in KRS-SCCs by comparing the percentage of CD4^+^CD25^+^ (Figure 6D) and CD4^+^ FoxP3^+^ population (Figure 6E) in TILs between wt and CD8^−/−^ recipients. Contrary to previous findings, we did not detect any significant difference in tumor infiltrating Tregs between wt and CD8^−/−^ recipients (Figure 6D and 6E). Therefore, we conclude that the presence of CD4^+^CD25^+^ and CD4^+^ FoxP3^+^ population is not dependent on CD8^+^ TILs. Taken together, our results demonstrate that the absence of CD8^+^ T cells leads to differential effects on distinct TIL populations.

### Lymphocyte-induced PD-L1 upregulation in KRS-SCCs

**Figure 7:**
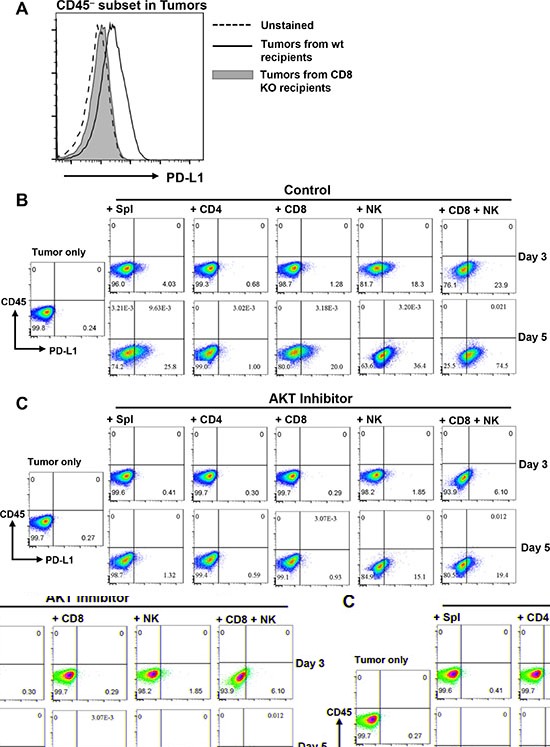
AKT-dependent lymphocyte-induced PD-L1 upregulation on SCCs (**A**) PD-L1 expression on CD45^−^ subset in tumors isolated from wt or CD8^−/−^ recipients as indicated. Data are representative results from 3 independent experiments. (**B**) KRS-SCC tumor cells were cultured either alone (Tumor only) or with wt B6 splenocytes (Spl), CD4^+^, CD8^+^, NK or CD8^+^ plus NK cells for 3 or 5 days. Cultured cells were harvested and examined for CD45 vs PD-L1 expression via flow cytometry. (**C**) KRS-SCC tumor cells were cultured either alone (Tumor only) or with different subsets of lymphocytes as described above in the presence of AKT inhibitor (GSK690693, 10 μM). (**D**) The presence of SMAD4 protein was confirmed in Smad4 expressing SCC line (K5/S2) by western blot with mouse splenocytes (Spl control) as positive control, KRS-SCC as negative control and β-actin as loading control. (**E**) Smad4 expressing SCC tumor cell line was cultured either alone (Tumor cell line only) or with wt B6 splenocytes (Tumor + Spl) for 5 days. Cultured cells were harvested and examined for CD45 vs PD-L1 expression via flow cytometry. Data are representative results from more than three independent experiments for panel B–E.

To further elucidate the immune evasion mechanisms of KRS-SCCs, we test whether these tumor cells can alter the expression of PD-L1 in response to lymphocytes. We found that KRS-SCC tumor cells isolated from wt recipients expressed a higher level of PD-L1 than those from CD8^−/−^ recipients (Figure 7A). Since tumor infiltrating NK cells were remarkably decreased in the KRS-SCCs from CD8^−/−^ recipients (Figure 6A and 6B), such tumor microenvironment not only lacked CD8^+^ T cells but also NK cells. Taken together, these data suggest that both CD8^+^ T cells and NK cells likely contribute to the upregulation of PD-L1 on KRS-SCCs *in vivo*.

To further dissect the mechanisms of lymphocyte-induced PD-L1 upregulation, we employed an *ex vivo* co-culturing assay by first testing whether wt B6 splenocytes can induce PD-L1 expression on tumor cells. Our data showed that KRS-SCCs indeed upregulated PD-L1 expression when co-cultured with wt B6 splenocytes in a time-dependent manner (Figure 7B, day3 and day5). Next, we isolated naïve CD4^+^, CD8^+^ or NK cells from wt B6 mice and co-cultured each population with KRS-SCCs individually. We found that CD8^+^ T cells or NK cells induced PD-L1 upregulation on KRS-SCCs whereas CD4^+^ T cells did not (Figure 7B). The effects of NK cells on PD-L1 upregulation appeared to be more rapid and profound than CD8^+^T cells (Figure 7B), and the combinatorial effects of both populations resulted in the most upregulation of PD-L1 on tumor cells (Figure 7B).

Previous studies identified the involvement of Pten-PI3K-AKT pathway in regulating PD-L1 expression in glioma or non-small cell lung cancers [39, 40]. Smad4 mutant SCCs of the skin exhibited the activation of AKT [9]. Thus, we tested whether lymphocyte-induced PD-L1 upregulation on KRS-SCCs was dependent on AKT signaling. To do so, we co-cultured KRS-SCCs with different subsets of lymphocytes as described above in the presence of AKT inhibitor. Our results showed that AKT inhibition significantly reduced the level of PD-L1 upregulation on KRS-SCCs induced by wt splenocytes, NK, CD8^+^ T cells, or NK plus CD8^+^ T cells (Figure 7C).

We further examined whether the lymphocyte-induced upregulation of PD-L1 on tumor cells is dependent on *Smad4* deletion. To do so, we employed another Smad4 expressing SCC cell line (Figure 7D) in our co-culturing assay. Our data showed that PD-L1 expression was also upregulated in the Smad4-expressing SCCs when co-cultured with wt B6 splenocytes (Figure 7E).

### Dual blockade of PD-1 and LAG-3 inhibits the tumor growth of KRS-SCCs

**Figure 8:**
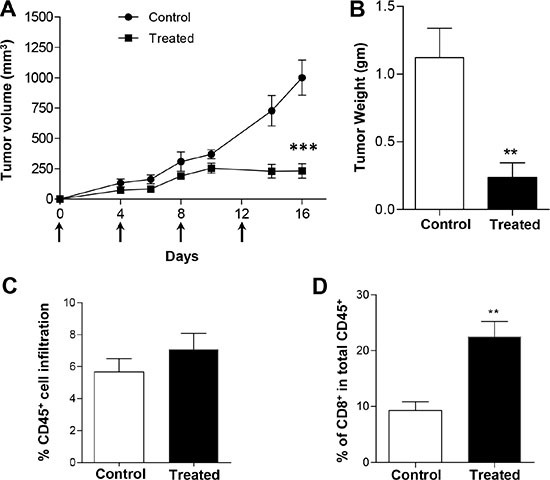
Dual blockade of PD-1 and LAG-3 inhibits the tumor growth of KRS-SCCs (**A**) KRS-SCC cells were injected into wt B6 recipients which were randomized into two groups. One group was treated with anti-PD-1 and anti-LAG-3 antibodies and another one was treated with PBS as control. Tumor volume was monitored in both control (closed circle, *n* = 8) and treated recipients (closed square, *n* = 8) for 16 days. Arrows indicated the injection of anti-PD-1 and anti-LAG-3 antibodies. (**B**) Tumor weight was measured at the end of the experiments from control (white bar, *n* = 8) or treated group (black bar, *n* = 8). (**C**) Percentage of CD45^+^ cell infiltration in the tumors isolated from control (white bar, *n* = 8) or treated group (black bar, *n* = 8). (**D**) Percentage of CD8^+^ T cells in total CD45^+^ infiltrating cells in the tumors isolated from control (white bar, *n* = 8) or treated group (black bar, *n* = 8). Statistical significance was calculated with Student's *t-test* (Graphpad Version 5.01) between two groups as indicated, ***P ≤ 0.001, **P ≤ 0.01. Representative data are shown from three independent experiments for all panels.

Our data showed that majority of CD8^+^ TILs co-expressed PD-1 and LAG-3 (Figure 3F), which likely rendered CD8^+^ T cells incapable of mounting effective anti-tumor immune responses. In addition, PD-1 and LAG-3 were co-expressed in a relatively large fraction of CD4^+^ TILs compared with other controls (Figure 5E). Hence, we next examined whether double blockade of PD-1 and LAG-3 could inhibit the tumor growth of KRS-SCCs, which would suggest a functional rescue of exhausted TILs. Wt B6 mice were inoculated with KRS-SCCs, treated with anti-PD-1 and anti-LAG3 antibodies or PBS control, and tumor growth was monitored for 2–3 weeks. Our data showed that dual blockade of PD-1 and LAG-3 significantly inhibited the tumor growth in the treated group compared to control one (Figure 8A). Consistently, the tumor weight was also significantly reduced in the treated group (Figure 8B). While the percentage of CD45^+^ infiltrating cells was not significantly different between control and treated group (Figure 8C), we found that the percentage of CD8^+^ TILs was significantly increased in the treated group as compared to control one (Figure 8D).

## DISCUSSION

The goal of our current study is to uncover the mechanisms of immune evasion in SCCs. We employ a transplanted model to reveal the immune profiling of TILs within KRS-SCCs, and present four unexpected novel findings: (1) majority of CD8^+^ TILs co-expressed PD-1 and LAG-3 and a higher percentage of them also downregulated their TCRβ expression compared to other controls; (2) CD4^+^ TILs exhibited a preferential expansion of Treg population, which is independent of the presence of CD8^+^ T cells in KRS-SCC tumor microenvironment, contradictory to prior findings in melanoma [20]; (3) Surprisingly, CD8^+^ T cells are required for the enhanced recruitment of NK cells in KRS-SCCs; (4) Both NK and CD8^+^ T cells can upregulate PD-L1 on KRS-SCCs, albeit NK cells' effects appear to be more rapid and profound, furthermore, such lymphocyte-induced PD-L1 upregulation is AKT-dependent. Taken together, our findings reveal novel immune evasion mechanisms of SCCs, and suggest that immunosuppressive mechanisms operate in a cancer-type specific and context-dependent manner. These results may have important implications in targeted-immunotherapy of SCCs and beyond.

We find that the percentage of CD8^+^ TILs is relatively increased compared to that of CD4^+^ TILs, moreover, CD8^+^ TILs also upregulate activation markers. Previous studies have suggested that a higher level of CD8^+^ or CD4^+^ infiltration correlated with a better survival in HNSCC patients [41]. However, CD8^+^ TILs appear to be overly activated and experience exhaustion within KRS-SCCs, evidenced by predominant co-expression of PD-1 and LAG-3 inhibitory co-receptors. Consistently, these chronically activated and exhausted CD8^+^ TILs failed to control tumor growth in wt recipients; moreover, we did not find significant difference in tumor growth between wt and CD8^−/−^ recipients. Thus, our studies further strengthen the rational for cancer immunotherapy of SCCs [15, 42], in addition, these studies may have important implications in patient selection for targeted-immunotherapy. Among the currently tested cancer immunotherapy approaches, immune checkpoint blockade has gained much attention due to its potential in inducing durable responses in various different types of cancers [25, 28, 36]. In this regard, we observed no difference in CTLA-4 or TIM-3 expression between CD8^+^ TILs and other control groups, hence CTLA-4 antibody, ipilimumab, might not be an ideal therapeutic choice for SCCs harboring RAS mutations or *Smad4* deletion. In this regard, a large multiple-center trial showed that combined radiation and anti-CTLA-4 blockade (ipilimumab) did not result in significant clinical benefits in 799 prostate cancer patients [43]. Based on our data showing that CD8^+^ TILs in KRS-SCCs co-expressed PD-1 and LAG-3 but not CTLA-4, we suggest that evaluating SIP of TILs might be critical for patient selection of targeted immunotherapy. Presumably, if TILs do not express CTLA-4, it would not be surprising that such targeted therapy has no significant effects. Numerous studies have shown that immune checkpoint inhibitors such as CTLA-4 or PD-1 blockade are effective in different types of cancers; yet, the response rates remain at 20–30% [25, 28, 36]. Attempts have been made to utilize the expression level of PD-L1 as a biomarker to predict therapeutic responses in patients, which works well in some but not all types of cancers [44], thus, we suggest that characterizing SIP of TILs might serve as another biomarker to predict clinical responses to immune checkpoint inhibitors as shown previously [45].

In our SCC tumor model, CD8^+^ TILs not only expressed a high level of PD-1 or LAG-3 but also predominantly co-expressed both inhibitory receptors. PD-1 and LAG-3 co-expression has been previously reported in chronically stimulated CD8^+^ T cells or implicated in ovarian cancers [46, 47]. Prior studies have also shown a synergistic role of PD-1 and LAG-3 in suppressing T cell functions in the context of tumor immune evasion [48]. While LAG-3^−/−^PD-1^−/−^ mice developed autoimmunity due to lack of self-tolerance [48, 49], they showed markedly increased survival and clearance of transplanted B16 melanoma or MC38 colon carcinoma [48]. Consistently, dual inhibition of PD-1 and LAG-3 suppressed tumor growth more effectively than a single inhibitor in transplanted mouse models of Sa1N fibrosarcoma, MC38 or CT26-HER-2 colon carcinoma [48, 50]. Apart from CD8^+^ TILs, we also found increased CD4^+^PD-1^+^ and CD4^+^LAG-3^+^ subsets in TILs compared to other controls (Figure 5), albeit the extent of increase is less prominent than in CD8^+^ TILs. Dual blockade of PD-1 and LAG-3 significantly inhibited the tumor growth of KRS-SCCs, suggesting a functional relevance of their co-expression on TILs. The next steps would be to investigate whether the phenotypes we observed in mouse models are also applicable in clinical settings, moreover, to determine whether double blockade of PD-1/LAG-3 would be effective for treating human SCCs, at least for certain subsets of patients.

In KRS-SCC tumors, we observed a preferential expansion of Tregs in CD4^+^ TILs, which appeared to occur in the DLN-TR group already (Figure 4D and 4F, DLN-TR), suggesting the influence of tumor microenvironment not only on TILs but also on T cells in nearby lymphoid organs, probably by affecting antigen presentation or tolerance mechanisms [37, 38, 51]. The expansion of Tregs in CD4^+^ TILs might contribute to the immune evasion of SCCs via multiple mechanisms, because Tregs can inhibit CD8^+^ T cell-mediated tumor killing, directly kill antigen presenting cells or secrete a number of inhibitory factors which have immune suppressive effects [37]. Previous studies show that the recruitment of Tregs at tumor sites is dependent on CD8^+^ T cells in melanoma models [20]. In contrast, we observed no difference in tumor infiltrating Tregs between wt and CD8^−/−^ recipients, demonstrating that Treg expansion is independent of CD8^+^ T cells in KRS-SCC tumor microenvironment. These observations might be explained by the potential involvement of TGF-β, whose expression is increased in Smad4 mutant HNSCCs [6]. TGF-β has been shown to play a critical role in generating and maintaining the population of induced Tregs [52–54], thus, the expanded Tregs might be sustained by tumor-derived TGF-β. Taken together, our data support the notion that immune evasion mechanisms operate in a tumor-type specific and context-dependent manner, implicating the influence of intrinsic properties of tumor cells.

Interestingly, we observed a remarkable reduction in the percentage of tumor infiltrating NK cells in CD8^−/−^recipients compared with wt counterparts (Figure 6). To our knowledge, this is the first report presenting evidence that the presence of CD8^+^ T cells is required for promoting the infiltration of NK cells within tumors. In general, previous findings usually suggest that NK cells orchestrate CD8^+^ TIL responses [55, 56]. Thus, our studies reveal a previously unrecognized role of CD8^+^ T cells in temporal control of tumor infiltrating NK cells, albeit the underlying mechanisms remain to be determined. Furthermore, our data uncover a combinatorial effect of NK and CD8^+^ T cells on upregulating PD-L1 expression in SCCs. While our results are consistent with previous studies showing a role of CD8^+^ TILs in upregulating PD-L1 expression [20, 21], we unexpectedly identify a more rapid and profound effect of NK cells, thereby suggesting the contribution of both populations *in vivo*, likely via producing IFN-γ. IFN-γ is predominantly secreted by CD8^+^ TILs as an anti-tumor agent but it can drive PD-L1 expression on tumor cells ultimately leading to immune resistance [57, 58]. Overall, our data suggest that “Adaptive Resistance Mechanism of Immune Escape” [21] likely contributes to the immune evasion of KRS-SCCs.

Tumor infiltrating B cells are less well studied; however, they may well contribute to the anti-tumor effects of TILs [59, 60]. For instance, a recent study showed that tumor-infiltrating B cells can interact with T cells to control the progression of hepatocellular carcinoma [61]. In our KRS-SCC model, we found that the percentage of TIL B cells is relatively low, compared to other subsets including CD4^+^, CD8^+^ or NK cells; especially when compared to splenic B cell population, TIL B cells are rather negligible. It remains completely unknown what would be the “appropriate” percentage of B cells in TILs, which is also likely dependent on the characteristics of tumor cells and the tumor microenvironment. The relatively reduced percentage of TIL B cells might be caused by mechanisms that suppress B cell trafficking to tumors, or it remains possible that the infiltration of T cells precludes B cell migration to tumors.

## MATERIALS AND METHODS

### Generation of KRS-SCCs, cell culture and western blot

Primary KRS-SCCs were generated previously [11]. Briefly, a mouse model of SCC was generated by targeting two frequent genetic mutations in human SCCs, oncogene *Kras*^G12D^ activation and *Smad4* deletion, to mouse keratin 15–expressing (K15^+^) stem cells [11]. Mice were bred to contain the following alleles: a K15 promoter–driven Cre recombinase (K15.CrePR1), Smad4 with exon 8 flanked by Lox P sites (Smad4f/f), and a constitutively active Kras^G12D^ mutation (LSL-KrasG12D) [11]. Activation of these mutant alleles was achieved by application of RU486 [11]. Two primary KRS-SCCs were employed (A223 and B866) to generate tumor cell lines. Both tumors have been passaged *in vivo* and *in vitro*. During the passage of A223, two lines were established from lung and lymph node metastasis (H496 and H500). In total, three cell lines were employed in the current study (A223-H500, A223-H496 and B866). SCC tumor cells were maintained in DMEM medium supplemented with 10% fetal bovine serum, HEPES, antibiotic-Antimycotic (100×), and 2mM glutamine. Western blot was performed to confirm the absence of SMAD4 proteins in KRS-SCCs with anti-Smad4 antibody (clone 4G1C6, ThermoFisher) and anti-β-actin (clone C4, SCT) as loading control.

### Transplanted mouse models for KRS-SCCs

Wt C57BL/6 (B6) and CD8^−/−^ mice (4–6 weeks) were purchased from Jackson laboratories (Bar Harbor, Maine). Mice were maintained under specific pathogen-free conditions in the vivarium facility of University of Colorado Anschutz Medical Campus. Three KRS-SCC lines were injected subcutaneously at both flanks of recipient mice with total 0.5 × 10^6^ or 1 × 10^6^ cells in 100 μl DMEM. In brief, each individual cell line was injected into a group of recipient mice as an independent experiment, for instance, A223-H500 was injected into 6 recipient mice as one independent experiment, while B866 was injected into other 3 recipient mice as another independent experiment (see details in Supplemental Table 1). None of the tumor cell lines has been mixed or injected together. Tumor growth was monitored for 2–3 weeks. For the dual blockade of PD-1 and LAG-3 experiments, KRS-SCC lines were injected subcutaneously at both flanks of wt B6 recipient mice with total 0.5 × 10^6^ cells in 100 μl DMEM. Mice were randomly divided into two groups. One group was injected with anti-PD-1 and anti-LAG-3 antibodies (BioXcell, USA) and another group was treated with PBS as control. 200 μg of each antibody was intraperitoneally injected starting from the day of tumor inoculation, and four injections were administrated in total at a four-day interval. Tumor growth was monitored for 2–3 weeks, and the tumor weight was measured at the end of the experiments. Animal work was approved by the Institutional Animal Care and Use Committee of University of Colorado Anschutz Medical Campus (Aurora, CO).

### Tumor dissociation, flow cytometry and FACS antibodies

Tumor, inguinal draining lymph node (DLN) and spleen were isolated from tumor bearing mice while spleen only was isolated from wt B6 non-tumor bearing mice. Spleen was mechanically dissociated into single cell suspension, and red blood cells (RBC) were lysed using RBC lysing buffer (Sigma Aldrich, USA). Tumor weight was measured before dissociation. DLNs and tumors were washed with HBSS buffer. Tumors were minced with surgical blades into smaller pieces. Liberase DL (50μg/ml) was added to both minced tumors and DLNs and incubated at 37°C for 20 to 30 minutes. Liberase was neutralized with medium, and cells were RBC lysed, filtered with cell strainers, and processed for flow cytometry. Anti-mouse antibodies CD45 (clone 30-F11) and CD69 (clone H1 .2F3) were purchased from BD Biosciences; CD3e (clone 145-2C11) and PD-1 (clone RMP1-30) from eBioscience; CD4 (clone GK1.5), CD8a (clone 53-6.7), LAG-3 (clone C9B7W), PD-L1 (clone 10F.9G2), CD25 (clone PC61), FoxP3 (clone MF-14), IFN-γ (clone XMG1.2), CTLA-4 (clone UC10-4B9), TIM-3 (clone B8.2C12), NK1.1 (clone PK136), B220 (clone RA3-6B2), CD19 (clone 6D5) and CD49b (clone DX5) from Biolegend. For intracellular staining, BD Fix/Permeabilization buffer was used according to the manufacturer's instructions. For FoxP3 staining, Biolegend intracellular staining kit was used according to the manufacturer's instructions. For intracellular staining of IFN-γ, equal numbers of cells from different groups were cultured for 4–6 hours in the presence of BD GolgiStop (BD Biosciences, USA) and then harvested and stained for flow cytometry. CD45 gate was used to differentiate tumor infiltrating lymphocytes (CD45^+^) from other cell types (CD45^−^) within tumors. The percent of CD4^+^ or CD8^+^ T cells, NK and B cells was presented as the percentage of CD45^+^ cells. Data was acquired on BD Fortessa and analyzed with flowjo software V10 (FLOWJO, Orgeon, USA).

### Cell purification, *ex vivo* tumor and immune cell co-culture and invasion assay

For isolation of primary lymphocytes, spleens were harvested from wt B6 mice, and single cell suspension was prepared and used for isolation of different subsets including CD4^+^, CD8^+^ or NK cells (StemCell Mouse CD4, CD8, or NK cell isolation EASY kit) according to the manufacturer's instructions. Purity of isolated cells was confirmed by flow cytometry using the following antibodies: for CD8^+^ T cells with anti-CD3e and anti-CD8, CD4^+^ T cells with anti-CD3e and anti-CD4; and NK cells with anti-NK1.1 and anti-CD49b antibodies.

Wt B6 splenocytes were cultured in lymphocyte medium supplemented with IL-2 (1:4000 IU) (ThermoFisher) and IL-4 (5 ng/ml) (ThermoFisher). CD4, CD8 T cells and NK cells were cultured in lymphocyte medium supplemented with IL-2 only. KRS-SCC tumor cells (A223-H500) and wt B6 splenocytes or different subsets of lymphocytes were co-cultured for different time points (day 3 or day 5). Similarly, Smad4 expressing SCCs (K5/S2) were co-cultured with wt B6 splenocytes for 5 days. K5/S2 SCC cells were derived from a spontaneous SCC developed in K5.Smad2^−/−^ mice [7]. AKT inhibitor (GSK690693) was purchased from SelleckChem (Houston, TX) and used at the concentration of 10 μM. Cultured cells were collected and analyzed by flow cytometry for PD-L1 expression with tumor cells (CD45^−^) distinguished from lymphocytes (CD45^+^) using CD45 as a marker.

For invasion assay, cytoselect 24-well invasion assay kit was purchased (CellBiolab, USA). 3 × 10^4^ NK cells were seeded in the upper chamber while the lower chamber contained either medium, CD4^+^ or CD8^+^ T cells (9 × 10^4^). Lymphocyte medium supplemented with IL-2 was added in both upper and lower chambers. Invaded cells were collected from bottom chambers after 72 hours and counted with flow cytometry using NK1.1 and CD49b as markers.

## SUPPLEMENTARY MATERIALS



## References

[1] Alam M, Ratner D (2001). Cutaneous squamous-cell carcinoma. N Engl J Med.

[2] Jennings L, Schmults CD (2010). Management of high-risk cutaneous squamous cell carcinoma. J Clin Aesthet Dermatol.

[3] van der Schroeff JG, Evers LM, Boot AJ, Bos JL (1990). Ras oncogene mutations in basal cell carcinomas and squamous cell carcinomas of human skin. J Invest Dermatol.

[4] Boukamp P (2005). Non-melanoma skin cancer: what drives tumor development and progression?. Carcinogenesis.

[5] Leemans CR, Braakhuis BJ, Brakenhoff RH (2011). The molecular biology of head and neck cancer. Nat Rev Cancer.

[6] Bornstein S, White R, Malkoski S, Oka M, Han G, Cleaver T, Reh D, Andersen P, Gross N, Olson S (2009). Smad4 loss in mice causes spontaneous head and neck cancer with increased genomic instability and inflammation. J Clin Invest.

[7] Hoot KE, Lighthall J, Han G, Lu SL, Li A, Ju W, Kulesz-Martin M, Bottinger E, Wang XJ (2008). Keratinocyte-specific Smad2 ablation results in increased epithelial-mesenchymal transition during skin cancer formation and progression. J Clin Invest.

[8] White RA, Malkoski SP, Wang XJ (2010). TGFbeta signaling in head and neck squamous cell carcinoma. Oncogene.

[9] Qiao W, Li AG, Owens P, Xu X, Wang XJ, Deng CX (2006). Hair follicle defects and squamous cell carcinoma formation in Smad4 conditional knockout mouse skin. Oncogene.

[10] Teng Y, Sun AN, Pan XC, Yang G, Yang LL, Wang MR, Yang X (2006). Synergistic function of Smad4 and PTEN in suppressing forestomach squamous cell carcinoma in the mouse. Cancer Res.

[11] White RA, Neiman JM, Reddi A, Han G, Birlea S, Mitra D, Dionne L, Fernandez P, Murao K, Bian L, Keysar SB, Goldstein NB, Song N (2013). Epithelial stem cell mutations that promote squamous cell carcinoma metastasis. J Clin Invest.

[12] Zwald FO, Brown M (2011). Skin cancer in solid organ transplant recipients: advances in therapy and management: part I. Epidemiology of skin cancer in solid organ transplant recipients. J Am Acad Dermatol.

[13] Berg D, Otley CC (2002). Skin cancer in organ transplant recipients: Epidemiology, pathogenesis, and management. J Am Acad Dermatol.

[14] Vesely MD, Kershaw MH, Schreiber RD, Smyth MJ (2011). Natural innate and adaptive immunity to cancer. Annu Rev Immunol.

[15] Allen CT, Clavijo PE, Van Waes C, Chen Z (2015). Anti-Tumor Immunity in Head and Neck Cancer: Understanding the Evidence, How Tumors Escape and Immunotherapeutic Approaches. Cancers (Basel).

[16] Jie HB, Gildener-Leapman N, Li J, Srivastava RM, Gibson SP, Whiteside TL, Ferris RL (2013). Intratumoral regulatory T cells upregulate immunosuppressive molecules in head and neck cancer patients. Br J Cancer.

[17] Schaefer C, Kim GG, Albers A, Hoermann K, Myers EN, Whiteside TL (2005). Characteristics of CD4+CD25+ regulatory T cells in the peripheral circulation of patients with head and neck cancer. Br J Cancer.

[18] De Costa AM, Schuyler CA, Walker DD, Young MR (2012). Characterization of the evolution of immune phenotype during the development and progression of squamous cell carcinoma of the head and neck. Cancer Immunol Immunother.

[19] Lau KM, Cheng SH, Lo KW, Lee SA, Woo JK, van Hasselt CA, Lee SP, Rickinson AB, Ng MH (2007). Increase in circulating Foxp3+CD4+CD25(high) regulatory T cells in nasopharyngeal carcinoma patients. Br J Cancer.

[20] Spranger S, Spaapen RM, Zha Y, Williams J, Meng Y, Ha TT, Gajewski TF (2013). Up-regulation of PD-L1, IDO, and T(regs) in the melanoma tumor microenvironment is driven by CD8(+) T cells. Sci Transl Med.

[21] Taube JM, Anders RA, Young GD, Xu H, Sharma R, McMiller TL, Chen S, Klein AP, Pardoll DM, Topalian SL, Chen L (2012). Colocalization of inflammatory response with B7-h1 expression in human melanocytic lesions supports an adaptive resistance mechanism of immune escape. Sci Transl Med.

[22] Parker BS, Rautela J, Hertzog PJ (2016). Antitumour actions of interferons: implications for cancer therapy. Nat Rev Cancer.

[23] Swann JB, Smyth MJ (2007). Immune surveillance of tumors. J Clin Invest.

[24] Thomas GR, Chen Z, Oechsli MN, Hendler FJ, Van Waes C (1999). Decreased expression of CD80 is a marker for increased tumorigenicity in a new murine model of oral squamous-cell carcinoma. Int J Cancer.

[25] Adachi K, Tamada K (2015). Immune checkpoint blockade opens an avenue of cancer immunotherapy with a potent clinical efficacy. Cancer Sci.

[26] Ito A, Kondo S, Tada K, Kitano S (2015). Clinical Development of Immune Checkpoint Inhibitors. Biomed Res Int.

[27] Pardoll DM (2012). The blockade of immune checkpoints in cancer immunotherapy. Nat Rev Cancer.

[28] Postow MA, Callahan MK, Wolchok JD (2015). Immune Checkpoint Blockade in Cancer Therapy. J Clin Oncol.

[29] Ishida Y, Agata Y, Shibahara K, Honjo T (1992). Induced expression of PD-1, a novel member of the immunoglobulin gene superfamily, upon programmed cell death. EMBO J.

[30] Francisco LM, Sage PT, Sharpe AH (2010). The PD-1 pathway in tolerance and autoimmunity. Immunol Rev.

[31] Workman CJ, Vignali DA (2003). The CD4-related molecule, LAG-3 (CD223), regulates the expansion of activated T cells. Eur J Immunol.

[32] Workman CJ, Cauley LS, Kim IJ, Blackman MA, Woodland DL, Vignali DA (2004). Lymphocyte activation gene-3 (CD223) regulates the size of the expanding T cell population following antigen activation *in vivo*. J Immunol.

[33] Huang CT, Workman CJ, Flies D, Pan X, Marson AL, Zhou G, Hipkiss EL, Ravi S, Kowalski J, Levitsky HI, Powell JD, Pardoll DM, Drake CG (2004). Role of LAG-3 in regulatory T cells. Immunity.

[34] Grosso JF, Kelleher CC, Harris TJ, Maris CH, Hipkiss EL, De Marzo A, Anders R, Netto G, Getnet D, Bruno TC, Goldberg MV, Pardoll DM, Drake CG (2007). LAG-3 regulates CD8+ T cell accumulation and effector function in murine self- and tumor-tolerance systems. J Clin Invest.

[35] Ngiow SF, Teng MWL, Smyth MJ (2011). Prospects for TIM3-Targeted Antitumor Immunotherapy. Cancer Res.

[36] Ott PA, Hodi FS, Robert C (2013). CTLA-4 and PD-1/PD-L1 Blockade: New Immunotherapeutic Modalities with Durable Clinical Benefit in Melanoma Patients. Clin Cancer Res.

[37] Campbell DJ, Koch MA (2011). Phenotypical and functional specialization of FOXP3+ regulatory T cells. Nat Rev Immunol.

[38] Vignali DA, Collison LW, Workman CJ (2008). How regulatory T cells work. Nat Rev Immunol.

[39] Lastwika KJ, Wilson W, Li QK, Norris J, Xu H, Ghazarian SR, Kitagawa H, Kawabata S, Taube JM, Yao S, Liu LN, Gills JJ (2016). Control of PD-L1 Expression by Oncogenic Activation of the AKT-mTOR Pathway in Non-Small Cell Lung Cancer. Cancer Res.

[40] Parsa AT, Waldron JS, Panner A, Crane CA, Parney IF, Barry JJ, Cachola KE, Murray JC, Tihan T, Jensen MC, Mischel PS, Stokoe D, Pieper RO (2007). Loss of tumor suppressor PTEN function increases B7-H1 expression and immunoresistance in glioma. Nat Med.

[41] Nguyen N, Bellile E, Thomas D, McHugh J, Rozek L, Virani S, Peterson L, Carey TE, Walline H, Moyer J, Spector M, Perim D, Prince M (2016). Tumor infiltrating lymphocytes and survival in patients with head and neck squamous cell carcinoma. Head Neck.

[42] Ferris RL (2015). Immunology and Immunotherapy of Head and Neck Cancer. J Clin Oncol.

[43] Kwon ED, Drake CG, Scher HI, Fizazi K, Bossi A, van den Eertwegh AJ, Krainer M, Houede N, Santos R, Mahammedi H, Ng S, Maio M, Franke FA (2014). Ipilimumab versus placebo after radiotherapy in patients with metastatic castration-resistant prostate cancer that had progressed after docetaxel chemotherapy (CA184–043): a multicentre, randomised, double-blind, phase 3 trial. Lancet Oncol.

[44] Zou W, Wolchok JD, Chen L (2016). PD-L1 (B7-H1) and PD-1 pathway blockade for cancer therapy: Mechanisms, response biomarkers, and combinations. Sci Transl Med.

[45] Tumeh PC, Harview CL, Yearley JH, Shintaku IP, Taylor EJ, Robert L, Chmielowski B, Spasic M, Henry G, Ciobanu V, West AN, Carmona M, Kivork C (2014). PD-1 blockade induces responses by inhibiting adaptive immune resistance. Nature.

[46] Matsuzaki J, Gnjatic S, Mhawech-Fauceglia P, Beck A, Miller A, Tsuji T, Eppolito C, Qian F, Lele S, Shrikant P, Old LJ, Odunsi K (2010). Tumor-infiltrating NY-ESO-1–specific CD8(+) T cells are negatively regulated by LAG-3 and PD-1 in human ovarian cancer. Proc Natl Acad Sci USA.

[47] Grosso JF, Goldberg MV, Getnet D, Bruno TC, Yen H-R, Pyle KJ, Hipkiss E, Vignali DAA, Pardoll DM, Drake CG (2009). Functionally Distinct LAG-3 and PD-1 Subsets on Activated and Chronically Stimulated CD8 T Cells. J Immunol.

[48] Woo S-R, Turnis ME, Goldberg MV, Bankoti J, Selby M, Nirschl CJ, Bettini ML, Gravano D, Vogel P, Liu CL, Tangsombatvisit S, Grosso JF, Netto G (2012). Immune inhibitory molecules LAG-3 and PD-1 synergistically regulate T cell function to promote tumoral immune escape. Cancer Res.

[49] Okazaki T, Okazaki IM, Wang J, Sugiura D, Nakaki F, Yoshida T, Kato Y, Fagarasan S, Muramatsu M, Eto T, Hioki K, Honjo T (2011). PD-1 and LAG-3 inhibitory co-receptors act synergistically to prevent autoimmunity in mice. J Exp Med.

[50] Foy SP, Sennino B, dela Cruz T, Cote JJ, Gordon EJ, Kemp F, Xavier V, Franzusoff A, Rountree RB, Mandl SJ (2016). Poxvirus-Based Active Immunotherapy with PD-1 and LAG-3 Dual Immune Checkpoint Inhibition Overcomes Compensatory Immune Regulation, Yielding Complete Tumor Regression in Mice. PLoS ONE.

[51] Sakaguchi S, Yamaguchi T, Nomura T, Ono M (2008). Regulatory T Cells and Immune Tolerance. Cell.

[52] Fu S, Zhang N, Yopp AC, Chen D, Mao M, Chen D, Zhang H, Ding Y, Bromberg JS (2004). TGF-β Induces Foxp3 + T-Regulatory Cells from CD4 + CD25 − Precursors. Am J Transplant.

[53] Bird L (2010). Regulatory T cells: Nurtured by TGF[beta]. Nat Rev Immunol.

[54] Tran DQ (2012). TGF-β: the sword, the wand, and the shield of FOXP3+ regulatory T cells. J Mol Cell Biol.

[55] Fan Z, Yu P, Wang Y, Wang Y, Fu ML, Liu W, Sun Y, Fu YX (2006). NK-cell activation by LIGHT triggers tumor-specific CD8+ T-cell immunity to reject established tumors. Blood.

[56] Wong JL, Berk E, Edwards RP, Kalinski P (2013). IL-18-primed helper NK cells collaborate with dendritic cells to promote recruitment of effector CD8+ T cells to the tumor microenvironment. Cancer Res.

[57] Tsushima F, Tanaka K, Otsuki N, Youngnak P, Iwai H, Omura K, Azuma M (2006). Predominant expression of B7-H1 and its immunoregulatory roles in oral squamous cell carcinoma. Oral Oncol.

[58] Dong H, Strome SE, Salomao DR, Tamura H, Hirano F, Flies DB, Roche PC, Lu J, Zhu G, Tamada K, Lennon VA, Celis E, Chen L (2002). Tumor-associated B7-H1 promotes T-cell apoptosis: A potential mechanism of immune evasion. Nat Med.

[59] Nelson BH (2010). CD20+ B cells: the other tumor-infiltrating lymphocytes. J Immunol.

[60] Linnebacher M, Maletzki C (2012). Tumor-infiltrating B cells: The ignored players in tumor immunology. Oncoimmunology.

[61] Garnelo M, Tan A, Her Z, Yeong J, Lim CJ, Chen J, Lim KH, Weber A, Chow P, Chung A, Ooi LL, Toh HC, Heikenwalder M (2015). Interaction between tumour-infiltrating B cells and T cells controls the progression of hepatocellular carcinoma. Gut.

