# Supplementary material for: Squamous cell carcinomas escape immune surveillance via inducing chronic activation and exhaustion of CD8+ T Cells co-expressing PD-1 and LAG-3 inhibitory receptors
Source: Oncotarget. 2016 Nov 9;7(49):81341–56. doi: 10.18632/oncotarget.13228 (PMC5340255; doi:10.18632/oncotarget.13228)
Supplement: Supplementary file 1 [file oncotarget-07-81341-s001.pdf]

# Squamous cell carcinomas escape immune surveillance via inducing chronic activation and exhaustion of CD8$^{+}$ T cells co-expressing PD-1 and LAG-3 inhibitory receptors

## Supplementary Materials

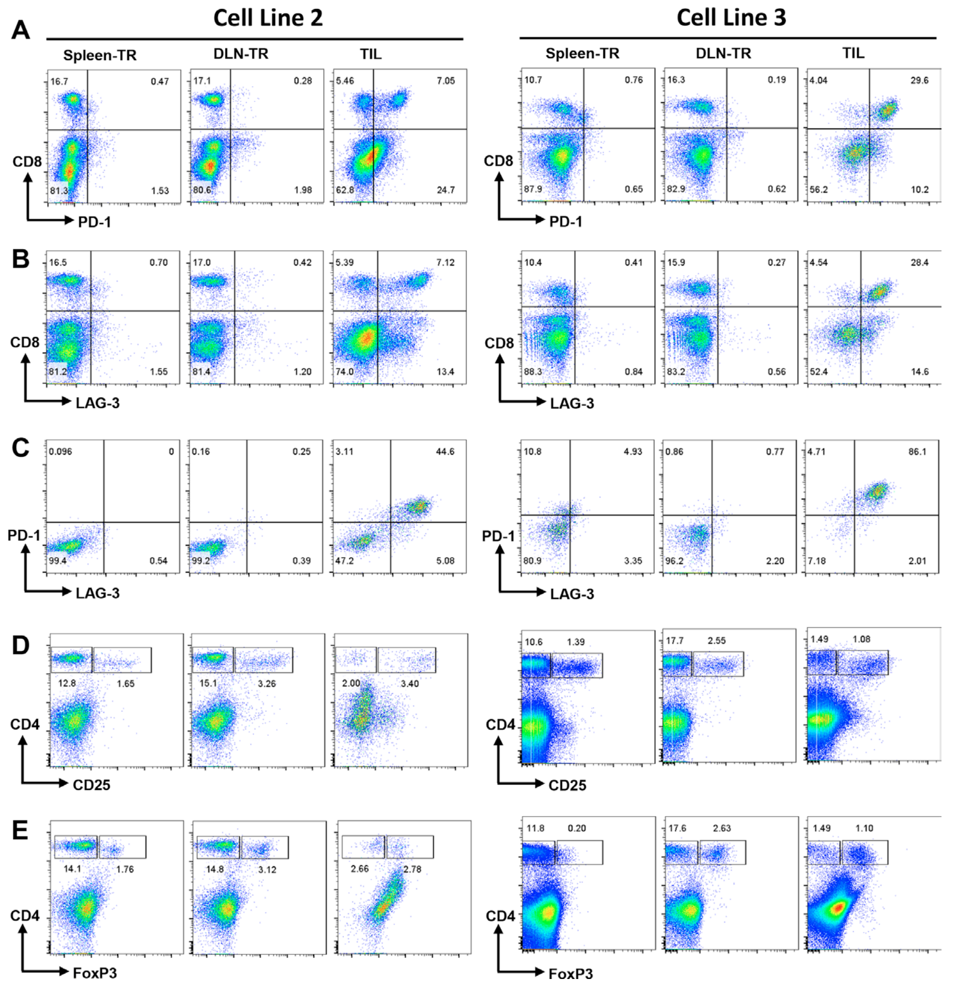


**Supplementary Figure S1: Immune profiling of TILs in additional KRS-SCC cell lines.** KRS-SCC cell line 2 (A223-H496) or cell line 3 (B866) were injected into wt B6 recipient mice individually to validate the immune phenotypes observed in cell line 1 (A223-H500). (**A**–**C**) Representative FACS plots are shown for CD8 vs PD-1 (A), CD8 vs LAG-3 (B), PD-1 vs LAG-3 gated on CD8$^{+}$ (**C**). (**D**–**E**) Representative FACS plots are shown for CD4 vs CD25 (D) or CD4 vs FoxP3 (E) staining in different groups.

**Supplemental Table S1: Summary of *in vivo* experiment replicates and sample size**

| Experiment | # of Experiments | Cell line | Sample |
|---|---|---|---|
| **TIL Profile** | Exp 1 | Cell line 1 | Tumor (*n* = 8) |
| | Exp 2 | Cell line 1 | Tumor (*n* = 6) |
| | Exp 3 | Cell line 1 | Tumor (*n* = 12) |
| | Exp 4 | Cell line 2 | Tumor (*n* = 8) |
| | Exp 5 | Cell line 3 | Tumor (*n* = 6) |
| | Exp 6 | Cell line 2 | Tumor (*n* = 6) |
| | Exp 7 | Cell line 1 | Tumor (*n* = 10) |
| **Wt vs CD8KO recipients** | Exp 1 | Cell line 1 | Control (*n* = 6), CD8KO (*n* = 6) |
| | Exp 2 | Cell line 1 | Control (*n* = 6), CD8KO (*n* = 6) |
| | Exp 3 | Cell line 1 | Control (*n* = 6), CD8KO (*n* = 6) |
| | Exp 4 | Cell line 1 | Control (*n* = 12), CD8KO (*n* = 12) |
| **Dual Inhibition** | Exp 1 | Cell line 1 | Control (*n* = 10), Treatment (*n* = 14) |
| | Exp 2 | Cell line 1 | Control (*n* = 8), Treatment (*n* = 8) |
| | Exp 3 | Cell line 1 | Control (*n* = 4), Treatment (*n* = 6) |
